# Air quality dataset from an indoor airport travelers transit area

**DOI:** 10.1016/j.dib.2023.109821

**Published:** 2023-11-19

**Authors:** Luca Davoli, Laura Belli, Gianluigi Ferrari

**Affiliations:** Internet of Things (IoT) Lab, Department of Engineering and Architecture, University of Parma, Parma 43124, Italy

**Keywords:** Indoor air quality, Gases concentration, Particulate matter (PM), CO_2_, Air temperature, Air humidity, COTS devices, COTS sensors

## Abstract

The experimental dataset (organized in a semicolon-separated text format) is composed by air quality records collected over a 1-year period (October 2022-October 2023) in an indoor travelers’ transit area in the Brindisi airport, Italy. In detail, the dataset consists of three CSV files (ranging from 7M records to 11M records) resulting from the on-field data collection performed by three prototypical *Internet of Things* (IoT) sensing nodes, designed and implemented at the IoTLab of the University of Parma, Italy, featuring a Raspberry Pi 4 (as processing unit) which three low-cost commercial sensors (namely: Adafruit MiCS5524, Sensirion SCD30, Sensirion SPS30) are connected to. The sensors sample the air in the monitored static indoor environment every 2 s. Each collected record composing the experimental dataset contains (i) the identifier of the IoT node that sampled the air parameters; (ii) the presence of gases (as a unified value concentration); (iii) the concentration of carbon dioxide (CO_2_) in the travelers’ transit area, together with air temperature and humidity; and (iv) the concentration of particulate matter (PM) in the indoor monitored environment – in terms of particles’ mass concentration (µg/m^3^), number of particles (#/cm^3^), and typical particle size (µm) – for particles with a diameter up to 0.5 µm (PM_0.5_), 1 µm (PM_1_), 2.5 µm (PM_2.5_), 4 µm (PM_4_), and 10 µm (PM_10_). Therefore, on the basis of the monitored air parameters in the indoor travelers’ transit area, the experimental dataset might be expedient for further analyses – e.g., for calculating Air Quality Indexes (AQIs) taking into account the collected information – and for comparison with information sampled in different contexts and scenarios – examples could be indoor domestic environments, as well as outdoor monitoring in smart cities or public transports.

Specifications Table

**Table:** 

Subject	Data Science / Pollution
Specific subject area	Dataset (i.e., gases’ concentrations, CO_2_ concentration, air temperature and humidity, PM) collected in the travelers’ transit area of an Italian airport (in the context of the EU project InSecTT)
Data format	Raw
Type of data	Table
Data collection	The experimental data collection lasted 1 year (from October 2022 to October 2023), during which different air parameters have been sampled (every 2 s) in the indoor travelers’ transit area of the Brindisi airport, Italy, via three prototypical IoT devices. In detail, each IoT node, equipped with three low-cost commercial sensors (namely: Adafruit MiCS5524, Sensirion SCD30, Sensirion SPS30) detecting gases concentration, CO_2_ concentration, air temperature and humidity, and PMs levels, has been deployed on the ceiling of the transit area, in three different positions. Each IoT node samples the air, checks for anomalous values (e.g., due to sensors reading errors), and store the collected data in a local (at the airport premises) MySQL database.
Data source location	The experimental data have been collected at the “Aeroporto del Salento” located in Contrada Baroncino, 72100 Brindisi, Italy (approx. latitude: 40.65832, approx. longitude: 17.93961).
Data accessibility	Repository name: “Indoor Air Quality Monitoring @ Brindisi Airport” Data identification number: doi:10.17632/bv2hvm4pmz Direct URL to data: https://doi.org/10.17632/bv2hvm4pmz

## 1 Value of the Data


 

• These experimental data are useful to estimate how the air quality may vary during the day on the basis of the variable rate of travelers passing by an indoor transit area inside an airport, allowing the detection of trends of interest [1].
• Researchers, program managers, operators and airport administrators may benefit from this experimental dataset through the application of various analysis techniques (e.g., based on statistical methods, as well as involving Machine Learning, ML, and Deep Learning, DL, algorithms) on both single air parameters, as well as considering a combination of multiple air quality data, looking for correlations of interest [2].
• Researchers can re-use the data contained in this dataset by comparing them with information collected in similar indoor contexts and scenarios (e.g., public airports, domestic homes, offices, and workplaces), as well as with data sampled in outdoor environments, in order to estimate the variability of the air quality [3].
• These data can be analyzed through statistical techniques, as well as algorithms based on Machine Learning (ML) and Deep Learning (DL) [4].

## 2 Data Description

This article describes the dataset [5], collected in the context of the European project InSecTT [6], containing air quality parameters sampled inside the travelers’ transit area in the “Aeroporto del Salento” airport [7] in the city of Brindisi, in the south of Italy. In detail, the dataset consists of three separated CSV files, each one denoted as complete_measure_node_airport_COLOR.csv and associated with a corresponding prototypical IoT node that effectively sampled and collected the data, and contains the information listed in Table 1, separated with semicolons.

**Table 1:** Content of each CSV file composing the dataset.

Column	Title	Parameter Content
0	id_measure	Unique identifier of the record (as integer value)
1	node_id	Textual identifier of the sampling IoT device
2	ads1115_value	Gases concentration value (as an aggregate value) returned by the MiCS5524 sensor
3	ads1115_voltage	Voltage value corresponding to the gases concentration identified by the “ads1115_value” field
4	scd30_co2	CO_2_ concentration, expressed as parts per million (ppm)
5	scd30_temp	Air temperature, expressed as Celsius degrees (°C)
6	scd30_hum	Air humidity, expressed as percentage per relative humidity (%RH)
7	sps30_pm05_count	Spatial concentration (expressed as #/cm^3^) of particles with a diameter up to 0.5 µm (PM_0.5_)
8	sps30_pm1_count	Spatial concentration (expressed as #/cm^3^) of particles with a diameter up to 1 µm (PM_1_)
9	sps30_pm1_ug	Mass concentration (expressed as µg/m^3^) of particles with a diameter up to 1 µm (PM_1_)
10	sps30_pm25_count	Spatial concentration (expressed as #/cm^3^) of particles with a diameter up to 2.5 µm (PM_2.5_)
11	sps30_pm25_ug	Mass concentration (expressed as µg/m^3^) of particles with a diameter up to 2.5 µm (PM_2.5_)
12	sps30_pm4_count	Spatial concentration (expressed as #/cm^3^) of particles with a diameter up to 4 µm (PM_4_)
13	sps30_pm4_ug	Mass concentration (expressed as µg/m^3^) of particles with a diameter up to 4 µm (PM_4_)
14	sps30_pm10_count	Spatial concentration (expressed as #/cm^3^) of particles with a diameter up to 10 µm (PM_10_)
15	sps30_pm10_ug	Mass concentration (expressed as µg/m^3^) of particles with a diameter up to 10 µm (PM_10_)
16	sps30_pm_typ	Typical particle size (expressed as µm) of the PM
17	ts_insertion	Timestamp (in Unix format) of the data sampling's time instant

More in detail, with regard to the parameters sampled by the MiCS5524 sensor [8], the gases detectable by this sensor (in the end resulting as a unique value, since the sensor cannot return each single gas concentration) are the following: carbon monoxide (CO), in the range 1 – 1,000 ppm; ethanol (C_2_H_6_OH), in the range 10 – 500 ppm; hydrogen (H_2_), in the range 1 – 1,000 ppm; ammonia (NH_3_), in the range 1 – 500 ppm; methane (CH_4_), for concentrations greater than 1,000 ppm. Referring to the SCD30 sensor [9], it can detect: CO_2_ in the range 0 – 40,000 ppm; air temperature in the range -40°C – 70°C; and air humidity in the range 0 %RH – 100 %RH. Finally, regarding the SPS30 sensor [10], it can detect particles with (i) a mass concentration in the range 0 – 1,000 µg/m^3^, (ii) a spatial concentration in the range 0 – 3,000 #/cm^3^, and (iii) a particle size ranging from 0.3 µm to 10 µm.

Finally, for the sake of clarity, the association with a generic color name (regarding the CSV files name) has been a consequence of an anonymization naming process for the IoT nodes, in order to hide their precise positions inside the transit area, being the airport spaces usually critical areas for safety and security reasons (especially those airside).

## 3 Experimental Design, Materials and Methods

The experimental data sampling has been performed over a 1-year period (from October 2022 to October 2023) in an indoor travelers’ transit area in the Brindisi airport, Italy, through the deployment of three prototypical IoT sensing nodes designed at the IoTLab of the University of Parma (https://iotlab.unipr.it). Each air quality monitoring device features a Raspberry Pi 4 (RPi4) [11] Single Board Computer (SBC) as processing unit, and the three low-cost commercial sensors (namely, Adafruit MiCS5524, Sensirion SCD30, Sensirion SPS30).

More in detail, each sensor was connected to the RPi4 via the I^2^C pins available on the SBC (with the MiCS5524 requiring an analog-to-digital converter), then powered by means of the power pins based on their specific requirements. Once the hardware deployment was completed, a software script, running inside each RPi4, carries out the following operational steps:

1. it opens (with proper connection parameters) and verifies the serial connection to each sensor equipping the IoT device;
2. it runs a “cleaning” procedure on the SPS30 sensor through the fan internal to the sensor itself, in order to clean from any residual dirt;
3. it runs a cyclic loop featuring a data querying on each sensor, in order to collect the last air quality parameters values, with the loop being executed every 2 s;
4. it verifies the validity of the values (i.e., due to reading errors on the I^2^C bus) and stores the collected data in a SQL-based database.

With regard to the indoor location where the IoT nodes have been deployed in the monitored environment, the order in which travelers pass by these IoT devices is the following:

node-air-gold→node-air-silver→node-air-brown with every prototypical sensing node located at a 3 m height.

## Limitations

The dataset has been collected over a 1-year period in an internal area of the airport with prototypical IoT devices that sometimes, likely because of software faults, might have stopped working. Therefore, some gaps in the collected data series are possible. Finally, the dataset can be considered as representative for a medium-sized indoor travelers’ transit area located on the airside of a medium-sized airport, where thousands of people pass by every day. In order to make the dataset cover an entire airport, additional IoT devices should be deployed in other (airside and landside) areas.

## Ethics Statement

We have read and follow the ethical requirements for publication in Data in Brief, and we confirm that the current work does not involve human subjects, animal experiments, or any data collected from social media platforms.

## CRediT authorship contribution statement

**Luca Davoli:** Conceptualization, Methodology, Software, Validation, Formal analysis, Investigation, Resources, Data curation, Writing – original draft, Writing – review & editing, Visualization. **Laura Belli:** Conceptualization, Methodology, Software, Validation, Formal analysis, Investigation, Resources, Data curation, Writing – review & editing, Visualization. **Gianluigi Ferrari:** Conceptualization, Methodology, Validation, Formal analysis, Resources, Writing – review & editing, Supervision, Project administration, Funding acquisition.

## Data Availability

Indoor Air Quality Monitoring @ Brindisi Airport (Original data) (Mendeley Data).
